# Information sought, information shared: exploring performance and image enhancing drug user-facilitated harm reduction information in online forums

**DOI:** 10.1186/s12954-017-0176-8

**Published:** 2017-07-21

**Authors:** Boden Tighe, Matthew Dunn, Fiona H. McKay, Timothy Piatkowski

**Affiliations:** 1 School of Health and Social Development, Deakin University, Geelong, Australia; 2 National Drug and Alcohol Research Centre, University of New South Wales, Sydney, New South Wales Australia; 3 Queensland University of Technology, Brisbane, Australia; 4 Centre for Youth Substance Abuse Research (CYSAR), Brisbane, Australia; 5 School of Health and Social Development, Geelong Waterfront Campus, Locked Bag 20000, Geelong, Victoria 3220 Australia

**Keywords:** Steroids, Online forums, Harm reduction, Anabolic-androgenic steroids, Internet forum

## Abstract

**Background:**

There is good evidence to suggest that performance and image enhancing drug (PIED) use is increasing in Australia and that there is an increase in those using PIEDs who have never used another illicit substance. Peers have always been an important source of information in this group, though the rise of the Internet, and the increased use of Internet forums amongst substance consumers to share harm reduction information, means that PIED users may have access to a large array of views and opinions. The aim of this study was to explore the type of information that PIED users seek and share on these forums.

**Methods:**

An online search was conducted to identify online forums that discussed PIED use. Three discussion forums were included in this study: aussiegymjunkies.com, bodybuildingforums.com.au, and brotherhoodofpain.com. The primary source of data for this study was the ‘threads’ from the online forums. Threads were thematically analysed for overall content, leading to the identification of themes.

**Results:**

One hundred thirty-four threads and 1716 individual posts from 450 unique avatars were included in this analysis. Two themes were identified: (1) personal experiences and advice and (2) referral to services and referral to the scientific literature.

**Conclusions:**

Internet forums are an accessible way for members of the PIED community to seek and share information to reduce the harms associated with PIED use. Forum members show concern for both their own and others’ use and, where they lack information, will recommend seeking information from medical professionals. Anecdotal evidence is given high credence though the findings from the scientific literature are used to support opinions. The engagement of health professionals within forums could prove a useful strategy for engaging with this population to provide harm reduction interventions, particularly as forum members are clearly seeking further reliable information, and peers may act as a conduit between users and the health and medical profession.

## Background

The Internet has become an important source of information about a range of health issues [1, 2]. In addition to being a platform from which people can seek information, it allows for the sharing of personalised accounts through online forums [3]. The growth of personalised feedback through these forums has been said to transform consumers into reflexive researchers who make active decisions based on reviews and peer-led insight [4, 5]. Online forums in particular are important avenues for knowledge exchange for those engaging in illicit or stigmatising behaviours, as they allow for anonymity while also affording a sense of community for those who participate [4, 6]. Online forums have led to a rise in peer-to-peer education and knowledge sharing and have created opportunities for users to engage with content by allowing members to contribute information.

Peers and social networks are an important component of the performance and image enhancing drug (PIED) community. Past research has demonstrated the importance these networks play in the supply of PIEDS [7–9], the distribution of injecting equipment [10, 11], and the sharing of advice and information about use [12–14]. Information was shared through networks, such as friends or fitness trainers, or physical documents such as fitness magazines, underground steroid manuals, and the scientific literature [15, 16]. Face-to-face interactions followed a strict social protocol that relied heavily on establishing trust and often took place in the public domain, for example in gyms [15, 16]. Links into these networks need to be made; usually some form of friendship is needed to facilitate engagement with the PIED-using community, and a new potential member must demonstrate a level of cultural knowledge to be accepted [8]. Socialisation into the group is an important part of the process [14].

However, the Internet has shifted the way those who consume substances seek and share information [17]. The Internet allows users to gain information from a large number of people without the need for potentially risky and identifying face-to-face interactions [1, 2]. Peer-led online forums in particular have been identified as common sources of information for PIED users [18] who report accessing Internet forums frequently to anonymously gain specific and detailed responses from other forum members about PIED use [19]. Furthermore, the first-hand information posted by anabolic-androgenic steroid (AAS) ‘veterans’ has been described as highly valued and is a preferred source for some who struggle to find accessible, straightforward information from reputable sources [20].

As information has shifted to online communities, there has been an increased research focus on the use of online forums by those who engage in substance use. Davey et al. [21] explored the key characteristics of online drug forums, finding that forums can provide users with anonymity, a space to communicate with others who are engaging the same, largely illicit, behaviour that is free from geographical limitations, and a dedicated place to share information with a community of individuals with similar interests. The notion that forums and forum members are a community has similarly been identified by others [6, 22, 23]. Forum members often possess a strong identity and group cohesion, strengthened by a sense of shared experience. Drug-related forums can also function as an avenue for social support, as well as advice mechanisms for crises, such as overdoses. Knowledge exchange, particularly regarding harm reduction practices, is a key feature [6, 23, 24], with users providing what has been described as ‘lay person evaluations’ of the risks and benefits of use [25].

The present literature suggests that online forums provide an environment for information and knowledge exchange. They are seen as supportive environments, refining a user’s social identity and social capital through communal processes. The scant research that has been conducted on PIED forums, however, suggests that image (especially muscularity) is viewed and associated with information status [26]––that is, those who are more muscular have their information privileged above that of others––something not seen in forums related to other substances, potentially making PIED forums a unique environment. Further, the harms related to PIED use largely differ from those of other substances, and as such, the information and knowledge exchange on dedicated PIED forums may differ. The aim of this study was to explore and characterise discussions regarding PIED-related harm on dedicated online forums.

## Methods

### Data collection

An online search was conducted using Google.com.au to identify online forums that discussed PIED use. Australian-based online forums were identified as ideal for this research as PIED use has increased in Australia in the past 6 years [27], and while there are similarities in PIED language across cultures, local terminology and slang are contextual and working with data from a single country limits opportunities for misinterpretation. It is acknowledged, however, that a key feature of online forums is that they are without borders [21], and people from any country can post in any forum they wish; indeed, research has shown that many forum users are members of multiple forums [22]. As such, it is not necessarily the case that the forums identified are truly ‘Australian’.

Search terms included ‘Australian bodybuilding forums’, ‘steroid chat rooms Australia’, and ‘performance-enhancing drugs Australia’. Sites were chosen on the basis of their Google search ranking. Only the first three pages of Google.com.au search results were included in the forum search, as previous studies suggest that 50% of users only view the first page and only 10% go beyond the third page [28]. To be included in the study, sites were required to be moderated, have a forum that was regularly used (i.e. daily posts by users), be publicly accessible and searchable, and be open to PIED use (demonstrating some sort of advice, education, and/or acknowledgement to health in using PIEDs). Sites were excluded if they were not Australian based, were largely news based, or predominantly served as product advertisement. Based on the inclusion and exclusion criteria, three online forums were included in this study: aussiegymjunkies.com, bodybuildingforums.com.au, and brotherhoodofpain.com.

**Fig. 1:**
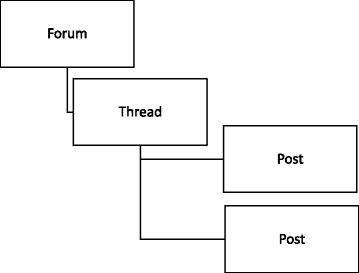
Threads consist of strings of posts that are connected by a central theme

The primary source of data for this study is the ‘threads’ from the online forums. Threads consist of strings of posts that are connected by a central theme. Threads can be considered to be discussions, with the structure of an online forum allowing for a community’s discussion history to be archived and searched and later retrieved [29] (see Fig. 1 for a visual depiction).

Depending on the structure of the forum, all threads from the main board or all threads from related sub-boards (e.g. anabolic steroids, steroids, and performance enhancers) were searched. The first five pages or first 50 threads (whichever was applicable, depending on how the forum site was designed) of the forum or sub-forum were collected for analysis in order of the most to least recent post. ‘Sticky threads’, permanently displayed threads that act as overarching review/general advice and do not typically allow forum members to post comments or discuss content, were excluded from the data. No other content restrictions were placed on threads; however, temporal limitations were applied, and any thread not commented on since 2012 was excluded, as data suggests that this is when a large increase in PIED users (especially novice users) attending needle and syringe programmes was detected [27]. Threads were read and downloaded between July and August, 2016. Following the method proposed by Hutchinson et al. [29], titles were read first, followed by (sub) threads. If the title directly or indirectly suggested PIED or AAS use, it was inspected. If there was any reference to PIED, the full thread was captured for analysis. Participation in the discussions on the website requires the creation of an individual avatar, which is used for identification between members. Each avatar has a ‘handle’, or nickname, and an individual may choose to reveal as much or as little about themselves as they wish, including a picture of themselves, their geographical location, age, and gender. Only handles were downloaded, though these have been excluded from quotes.

### Data analysis

Data analysis followed the five-step Empirical Phenomenological Psychological (EPP) approach used by Van Hout and Hearne [30]. The first step involved reading the dataset three times so as to become familiar with the threads, with the aim of developing an impartial overview in the complete absence of any specific hypothesis. The second step involved abbreviating the dataset into thematic responses without regard to syntax. The third step involved confirming the responses by collaborating with others in order to define the true understanding of the post. The fourth step involved analysing topical codes to determine a dominant theme. The fifth step involved categorising the codes into themes. Once identified, the threads were transferred to a Word document for analysis following the EPP protocol. The whole threads were also analysed for overall content. The threads were analysed following the constant comparative method advocated by Miles and Huberman [31]. The threads were read and re-read for content familiarity and to allow for the identification of these overarching themes. The themes were reviewed across forums and checked for distinction and coherence. Ethical approval was granted by the Deakin University Human Research Ethics Committee.

## Results

In total, 134 threads and 1716 individual posts from 450 unique avatars were included in this analysis. Threads as a whole were analysed to identify the types of information forum members sought and shared. Two overarching themes were identified from this analysis and are presented below and supported by presentative quotes (see also Table 1).

**Table 1:** Themes and topics characterised within posted thread responses

Themes	Topic codes
Discussion about personal experiences and advice and recommendations	Feedback based on personal experiences, including drug acquisition, substances, combinations, courses and durations, dose amounts, recovery reports, and even product evaluations. Recommendations based on drug administration, PIED combinations and contraindications, and course undertakings. Also incorporates advice or warnings about products, managing expectations or issues, and normalising experiences.
Referral to service and referring to the research	Involved specifically referring to a service (i.e. doctor, pathology, NSP) or forum member for advice. Also acknowledges the inquirer to read/research further information to attain better comprehension (i.e. read the stickies, search a specific URL).

### Theme 1: advice and personal experience

The first theme is concerned with the sharing of advice and personal experiences. Within this theme are examples of members sharing personal experience, information, tips, and knowledge to other, often more novice, members. Forum members shared information that came from their personal experiences and was not informed by evidence from the scientific literature. Shared anecdotes and experiences were both positive and negative in tone; information was rarely presented as fact, and members would often include qualifiers, such as pointing out that people responded differently to similar substances:

After my first long cycle (over 7 months) I came off for 12 whole months. Ran 50mg clomid (prescribed by urologist) for 6 months, then came off and took nothing for another 6. While on clomid the highest my test levels got to was 800. Then, when I stopped the clomid they went back down to the 300s.....this was after a full year. I'm not saying this is your case, but I think anyone who decides to take AAS has to realize they are potentially making a long term commitment.

A common element to posts within this theme was sharing ratings or reviews of products. Again, these reviews were based on personal experience and served to highlight to other forum members the different possible outcomes from using the same substance.

I highly rate this stack; don’t be surprised if you put on 5kgs of muscle. However, I would opt for a different ester.

Compared to my last course, this cycle was sh*t. Would look for a different compound to run if I was you.

While there was a bias toward discussing the positive effects of PIED use, such as large gains in size or strength, forum members also took the opportunity to describe their negative experiences or side effects from use. Given many of the forum members were engaging or seeking to engage in PIED use, the sharing of negative or less desirable side effects is important in providing novice users with a variety of possible outcomes.

I’m running this NPP Test stack too; night sweats are driving me insane, change track pants at midnight then wake drenched again.

The anonymity that forums provide allowed members to discuss important topics related to their experiences which may not come up in open forums. For example, discussions of negative experiences were not isolated to just physical effects. Members would openly discuss any negative mental health effects they were experiencing from particular substances or combinations of substances.

Member 1: If you are prone to anxiety don’t do it. It will f**k with your head.

Member 2: *I agree this stack put me in some dark places.*

The forums created the opportunity for disagreements and arguments between members. In these circumstances, members would use the ‘tagging’ function (@Members_Name) to include another member who they thought may have some useful input on the topic. Members who were included in these discussions were often seen as elders or as trusted advisers. Within these discussions, the opinions of more experienced members were both solicited and respected by both existing and newer members.

“Hey @… Can you tell this bloke why he shouldn’t be using insulin like he is?”

“@… knows his stuff”

“Check this sh*t out @… What do you recommend?”

Members asked for and were provided with specific practical advice in response to issues or concerns relating to the practicalities of PIED use. For example, members regularly sought advice about side effects, results, provision, course duration and type, or products, and in response, other members would offer specific instruction, advice, or recommendations. This advice was largely related to the mechanics of PIED use, with few value statements or attributions. In many of these comments, forum members guided other members in ways to prevent or minimise harm related either to the dose or course or to the side effects.

You should have done bloods earlier, but if your E2 is not excessively elevated there's no reason to take an AI prior to PCT now. Waiting for gyno symptoms isn't the best way to go either.

The didactical nature of the online forum meant that the advice given by some members was open to question and debate. Many of the debates were related to compound choice, dosage, timing, and reasoning. Given the wide range of options within PIED use, these discussions were frequently multisided and could get fairly heated between members who were of the opinion that their experience was more correct. For example:

Member 1: *“Increase dose by 50% and add in an entire new compound?” Can you please explain this logic to me? I mean I legitimately don't understand why you want to try something new?*

Member 2: *Bro, switching compounds is good; you can build tolerance to a compound. Go with it, no need to increase dosage amount though.*

Member 1: *You’re kidding. Compound tolerance? It’s his second course. Read the post. If I was you the most I would be looking at would be increasing the test dose alone, it doesn't sound like you need any additional compounds yet.*

Member 2: *Mate, I don't see any point in running the same compound. You’ve got to test the body to see what works. I've done five different compounds over five courses. Some people have compound tolerances and react better/worse to gear.*

Member 3: *Bro, if it ain’t broke, don’t fix it… Stick with what* [Member 1] *said. Go with just Test.*

Advice, recommendations, and assurance were also provided to assist forum members to manage distress or (unrealistic) expectations. In these situations, members provided comments to normalise unwanted or undesirable situations, thereby reassuring a newer member that they were not alone, or confirmed if the information under question was correct. For example, course and product combinations were promoted, with member choices reassured, or alternatives were suggested.

@… it looks pretty spot on mate. As long as your gear is all of good quality and you get bloods your gtg [good to go]*.*

Incorrect. Obviously longer esters like Test E are more beneficial for this cycle.

Experienced forum members (those with high post counts) often provided other members with guidance on their courses, particularly relating to the potency, dosage, and administration of products they were using. These threads were living and didactic, with members often coming back to revise their posts or to add more information to the thread, or with other members entering the discussion to provide more information or to ask questions. Members occasionally provided outside information, particularly in the form of Web addresses to complement their comment.

That amount of EQ will send your RBC through the roof and into outer space. Evidence has shown EQ to be effective at less than half your dose. See – [name of website]

Users openly discussed each other’s future courses, with specific information such as the compounds being used. They also made reference to contraindications, or possible harmful side effects, most likely with the intention of minimising harms to other users. Threads like these would generally become in-depth discussions involving the chemistry and pharmacology of specific compounds between forum members.

Though Ipamorelin is similar to other GHRP's such as GHRP-2 and GHRP-6. Ipamorelin does not cause sudden spikes in prolactin or cortisol like GHRP-2 and GHRP-6 can do.

### Theme 2: referral to services and referring to the scientific literature

Forum members often expressed an interest in increasing their knowledge and education about the substances they were using and how they could get the results they were seeking. This was complemented by more experienced members who made themselves available to share their own knowledge and experience. However, there were times when heated exchanges around a lack of knowledge occurred, often when there was a misunderstanding, or when one member felt that another member should seek external advice and not rely solely on the information provided by the forum. Much of this advice was of a harm reduction nature, for example in suggesting some post-cycle therapy or safer injecting practices. However, while many members were open with the advice they provided, they also acknowledged the limitations of their own knowledge or would openly admit that the only way an issue could be dealt with was to seek assistance from a general practitioner (GP), endocrinologist, or pathologist.

While forum members were prepared to share their own experience and tips, engagement with health professionals was recommended prior, during, and post-course. This positive role modelling from the more experienced users had the effect of encouraging novice users to seek information and to minimise potential harm. In addition to more traditional health care, ancillary services, including needle and syringe programmes (NSPs), pharmacists, and anti-ageing clinics, were also recommended by users as useful places to get information and supply of equipment.

Member 1: *Where on the Gold Coast can you pick up alcohol swabs and slin pins?*

Member 2: *Try the chemist on* [street name]*. If not, definitely the NSP.*

When it became clear to members that an inexperienced user did not understand or comprehend certain protocols, thread comments would refer them to ‘go and research’*.* Examples of this were seen in a number of comments that read ‘read the stickies’ (permanently displayed threads that act as overarching review/general advice and do not typically allow forum members to post comments or discuss content) or ‘refer to post/URL link’*.* Signs of aggression and frustration were perceived amongst these posts with users often commenting:

This is not a joke or something you want to f**k with.

Forum members and moderators appeared frustrated about the naivety of new users and their apparent disregard for their own personal safety. Blunt responses were common in addressing these users, with the suggestion that they further educate themselves.

Blast a bit of HCG with some zinc and folate, then get a semen analysis done to see where you at. There was a similar thread a month ago, search for that thread and have a read what you need to do and why.

Some members went so far as to direct members to scientific publications in their responses to newer forum members.

In short, yes low thyroid function (hypothyroidism) is associated with lower testosterone and elevated thyroid function (hyperthyroidism) with increased testosterone – See these articles to why [user lists four articles from the scientific literature]

## Discussion

The aim of this study was to explore and characterise discussions regarding PIED-related harm on dedicated online forums. An analysis of the forum posts and responses showed that forum members wanted to take a more active role in monitoring their health and understanding the risks of using AAS and other PIEDs. While there was a positive bias toward PIED use, members discussed the negative side effects, including mental health, and acknowledged the limitations of their own knowledge. These online communities operated similarly to real-world PIED communities, with clear hierarchies and the need to display cultural knowledge to be accepted as a member of the community. In line with the findings from previous studies of online drug forums, members visit these forums to learn how to avoid harm as well as help others use more safely, but their language may differ from traditional harm reduction discourses. This study highlights the importance of providing individuals with a range of opportunities to increase knowledge to decrease risky behaviours and any subsequent harm. While the accuracy of information was not investigated in this research, overall, the findings of this study highlight the Internet as a potentially useful source for individualised user support around harm reduction.

The findings of this study regarding peer support are consistent with the work of Marshall et al. [32] who suggest that seeking advice or feedback from peers about an illicit action is a form of harm reduction in itself. Almost one third of users sought course information in the form of member reviews, evaluations, or critiques on PIED combinations, course durations, and dose amounts. Peer-led support has been a widely adapted strategy in deterring harms, as this type of anecdotal information can provide a lived experience of drug use that holds weight with other members of that community [32, 33]. However, the illegal and stigmatised nature of PIED use encourages users to seek out anonymous online advice, which may not be of the highest quality. As noted by Brennan et al. [17], information exchanges contain a mixture of scientific and anecdotal evidence, with the latter appearing to be given higher credence; however, members acknowledged their own knowledge limitations and would refer the original poster to external real-world services. This demonstrates that members take the health of others––even those they have never and may never meet in real life––seriously and that they seek no vanity in posting potentially misleading information just for the sake of posting.

Forums have been found to be based on a clear hierarchy; some users are only members, while others function as moderators or administrators. Experienced members often have different privileges within the site itself and can have their advice, recommendations, and warnings trusted over those of others. Experienced members can display high calibre knowledge in the instances where incorrect, false, or hoax information is posted. In accordance with the results of other studies [6, 21–24], this study identified evidence of a hierarchy within the community of forum members. Experienced and ‘high-ranking’ members were often asked specifically to provide advice or had their opinions used to verify the accuracy of a response. This is consistent with research conducted by Smith and Stewart [26] into online discussion of doping, where members operated in a social hierarchy contingent on masculinity and pharmacological knowledge. These high-ranking members may serve as a conduit for providing harm reduction information to other less experienced members. However, the nature of forums means that a member may be ‘high ranking’ simply because they are a frequent poster, not because they possess specific or accurate information. Interestingly, the hierarchy and rules of the online community were consistent with what has been found in real-world PIED communities. Our analysis showed that members needed to display cultural knowledge before being accepted into the culture or be given specific advice from high-ranking and experienced members.

When experiential feedback, advice, fact, or recommendation could not be found in the thread, members frequently referred users to a specific health service or advised them to further seek health information. This type of advice was only identified in a small number of threads, suggesting that this response only occurs when users displayed a need for either a specific service (e.g. NSP), the behaviour has a high-risk consequence, or when poor understanding of the risks and harms of PIEDs is displayed. When discussions were oriented toward acute help and practical support, the majority of referrals involved that of health professionals or primary health services, such as GPs, endocrinologist, NSPs, or chemists, reinforcing the notion that forum users are engaging in an alternative form of harm reduction. While this may mean that forum members are being directed to appropriate medical care, past research has identified a high level of distrust by PIED users toward some in the health and medical professions [25]. Furthermore, some PIED users report difficulty accessing healthcare information [26], and some frontline healthcare workers report having an inadequate understanding of these substances and their harms [27]. While online social networks may be one way in which PIED users can access harm reduction information, there needs to be an effort to ensure that this information is accurate and evidence based.

One challenge for researchers investigating online forums is the difficulty in assessing the reliability and accuracy of the information, as much of the information is anecdotal, and not grounded in the scientific literature [21]. This may especially be the case for AAS and other PIEDs, as researchers have not investigated the effects of these substances at the doses that are commonly taken by this group. User accounts are often the only information available, and it has been suggested that user accounts––which are often detailed and rich––could act as a source of scientific data that is triangulated with other data sources to provide more insight into both positive and negative effects [6]. While these studies provide important insight into these communities, they may not be generalisable to communities who use PIEDs. For instance, one study into recreational drug forums found that there was some resistance to harm reduction information because pleasure seeking was prioritised [25]. This may not be a relevant feature of PIED forums. Peers, and their lived experiences, are valuable and highly sought after by others in that community, and the inclusion of health and medical professionals into PIED-related forums could provide an avenue for increasing the standard of care and strategies for addressing harm.

One important aspect of this research for health professionals is that forum users were not using health-related language in their discussions with each other nor were they were explicitly talking about ‘harm’ and ‘harm reduction’. Discussions by forum members regarding harm reduction were approached in other ways, for example through discussions of specific substances and which ones to avoid, course advice, and advice on safe injecting practices, as well as general safety tips. By working with these communities in their own language, health professionals may be able to play a role in reducing the traditional stigma that users perceive is directed toward them, while also providing medically sound information to support users in reducing harm and complementing user experience or anecdote commonly found on forums. In this instance, high-ranking peers may act as an important conduit between health and medical professionals and the PIED-using community.

There are limitations to the current study. Firstly, this study deliberately focused on Australian sites, with the expectation that this would allow for an investigation of the Australian context (e.g. NSPs, increased PIED use, and increases in novice users). However, it was evident that people from other countries were participating in the discussions, making this a not entirely Australian-specific study. Secondly, the discussions used for analysis in this study come from publically accessible websites and may not represent discussions which are occurring in private forums. Thirdly, the qualitative nature of the study means that there could be researcher bias in the analysis phase; however, the researchers were in constant communication throughout the analysis process, in an attempt to minimise any discrepancies with the analysis.

## Conclusions

Internet forums are an accessible way for members of the PIED community to seek and share information to reduce the harms associated with PIED use. Forum members show concern for both their own and others’ use and, where they lack information, will recommend seeking information from medical professionals. Anecdotal evidence is given high credence though the findings from the scientific literature are used to support opinions. The engagement of health professionals within forums could prove a useful strategy for engaging with this population to provide harm reduction interventions, particularly as forum members are clearly seeking further reliable information. Peers may act as a conduit between users and the health and medical profession. The information posted and shared in forums may be useful for health professionals and may be useful in documenting the course protocols that members report using, as well as identify support services for those experiencing harm or concerned about their PIED use.
